# Enhanced computerized cognitive remediation therapy improved cognitive function, negative symptoms, and GDNF in male long-term inpatients with schizophrenia

**DOI:** 10.3389/fpsyt.2024.1477285

**Published:** 2025-01-16

**Authors:** Peiyun Zhang, Lingyun Chen, Qianqian Qin, Chao Liu, Haijiao Zhu, Wenqing Hu, Xinyu He, Kaihong Tang, Qi Yan, Hongmei Shen

**Affiliations:** ^1^ Laboratory of Biological Psychiatry, Nantong Mental Health Center, Nantong Brain Hospital & Affiliated Mental Health Center of Nantong University, Nantong, China; ^2^ Key Laboratory of Neuroregeneration of Jiangsu and Ministry of Education, Co-innovation Center of Neuroregeneration, Nantong University, Nantong, China; ^3^ Department of Psychology, University of California, Davis, Davis, CA, United States

**Keywords:** schizophrenia, computerized cognitive remediation therapy, negative symptoms, cognitive impairments, brain-derived neurotrophic factor, glial cell line-derived neurotrophic factor

## Abstract

**Objective:**

Negative and cognitive symptoms present significant challenges in patients with schizophrenia, and cognitive remediation is a promising approach to alleviate these symptoms. This study aimed to explore the efficacy of computerized cognitive remediation therapy (CCRT) on psychiatric symptoms, cognitive deficits, and serum levels of brain-derived neurotrophic factor (BDNF) and glial cell line-derived neurotrophic factor (GDNF) in patients with schizophrenia.

**Materials and methods:**

Forty male long-term institutionalized inpatients with schizophrenia were assigned to either a CCRT group (n = 20) or a control group (n = 20). The CCRT intervention consisted of 40 individual 40-min sessions over 8 weeks, conducted five times a week. Psychiatric symptoms, cognition, and serum levels of BDNF and GDNF were assessed at baseline, 4 weeks, and 8 weeks.

**Results:**

Compared to the control group, the CCRT group exhibited decreased total Positive and Negative Syndrome Scale and negative subscale scores, as well as increased Montreal Cognitive Assessment and Repeatable Battery for the Assessment of Neuropsychological Status scores. Moreover, improvements in list recall were associated with reduced negative symptoms. Additionally, CCRT ameliorated the decrease in serum GDNF levels in patients with schizophrenia.

**Conclusion:**

The effectiveness of CCRT in alleviating negative symptoms was associated with improvements in list recall, and GDNF may play a role in the observed effects of CCRT in patients with schizophrenia.

## Introduction

Schizophrenia is a chronic, progressive disorder affecting 0.4-1% of the global population, impacting approximately 21 million people worldwide (1). This debilitating condition imposes a substantial burden on patients, families and communities (2, 3). The most florid manifestation of schizophrenia includes both positive and negative psychotic symptoms (4). Cognitive impairment is a core feature of schizophrenia, contributing significantly to functional disability (5–8). Although the current antipsychotics have effectively controlled the positive symptoms in most patients (8), alleviation of negative and cognitive symptoms is a particularly challenging area in schizophrenia as their existence is related to a significant long-term morbidity, poor functional outcomes and a high disability rate (8–11).

Negative symptoms and cognitive impairment share the same biological and pathological mechanisms in schizophrenia, and it has been reported that cognitive impairment is related to negative symptoms (12). Cognitive remediation (CR) has been recommended for the treatment of cognitive impairment, and negative symptoms have also been proposed as a new target for CR (13–16). The meta-analysis report stated that CR therapy (CRT), an evidence-based intervention, has beneficial effects on cognitive impairment and negative symptoms, and should be included in clinical guidelines for treating patients with schizophrenia (17, 18). However, regression modeling did not indicate any improvement in the performance of CR on neuropsychological composites (episodic memory, working memory, attention, executive functioning, processing speed), or functioning proxies (instrumental activities of daily living and interpersonal effectiveness) though it showed significant improvement on all but two of the 10 exercise metrics of CRT (19–21). Additionally, research has not yet examined which aspects of cognitive improvement by CRT are associated with the improvement of negative symptoms in schizophrenia.

The neurodevelopmental aberrations influenced by neurotrophic factors play a crucial role in the pathogenesis of schizophrenia. Glial cell line-derived neurotrophic factor (GDNF) and brain-derived neurotrophic factor (BDNF) are two widely studied families of neurotrophins in schizophrenia (22, 23). Compared with the healthy controls, peripheral BDNF and GDNF serum levels were significantly reduced in patients with schizophrenia (24, 25). BDNF has been proposed as a potential biomarker for schizophrenia, particularly in cognitive recovery (26). To our knowledge, a study tested BDNF as a peripheral biomarker for CRT-specific effects, but found no correlation between changes in BDNF levels and cognitive improvement in patients with schizophrenia (27). To date, no other studies have replicated these findings, although CRT has been shown to improve cognitive function in patients with schizophrenia (26, 28, 29). GDNF is important for dopaminergic neurons, and plays a critical role in the pathophysiology of schizophrenia (30, 31). However, studies on peripheral GDNF levels in patients with schizophrenia have shown inconsistent results (23, 32). There have been no previous studies exploring the effect of CRT on GDNF levels in patients with schizophrenia, leaving the relationship between serum neurotrophic factors and CRT unclear.

The primary objective of this study was to verify the improvement of computerized CRT (CCRT) on psychiatric symptoms and cognitive impairments in male long-term institutionalized inpatients with schizophrenia. We also explored the association between changes in cognitive aspects and psychiatric symptoms, as well as whether CCRT affected neurotrophic factors such as GDNF or BDNF. We hypothesized that patients with schizophrenia receiving CCRT would show significant improvement in psychiatric symptoms and cognitive functioning, and aimed to determine whether CCRT influenced neurotrophic factors to further understand their roles in schizophrenia.

## Materials and methods

This work was a quasi-randomized controlled trial to verify the effects of CCRT on cognitive functioning and psychiatric symptoms of inpatients with schizophrenia at Nantong Mental Health Center. This study has been registered on the National Health Security Information Platform in China (No.: MR-32-23-008913), and approved by the Ethics Committee of the Nantong Fourth People’s Hospital (No.: 2019-K013). All participants and their guardians have given written informed consent to participate in this study and publish the results.

### Participants

124 male subjects diagnosed with schizophrenia, based on the criteria of Diagnostic and Statistical Manual of Mental Disorders, 5^th^ edition (DSM-V) by two psychiatrists independently, were recruited from July 1, 2019 to June 30, 2021 at Nantong Mental Health Center. A total of 40 long-term institutionalized inpatients aged between 18 and 60 years who had been clinically stable for at least 6 months under antipsychotic medication were enrolled in this study. In addition, their PANSS scores, judged by two psychiatrists independently, remained unchanged in two consecutive assessments (once a month) prior to this study. The consolidated standards of reporting trials (CONSORT) flow diagram were shown in **Supplementary Figure 1**. Randomization was independently conducted by a nurse who did not participate in this study when the 40 inpatients were successfully enrolled. The online random number producer was used to generate a random number table consisting of 40 lots, each of which was drawn into a sealed envelope and assigned to each patient. Each patient was randomly assigned a unique identifier. Participants with odd-numbered identifiers were assigned to the control group, while those with even-numbered identifiers were assigned to the CCRT group. The patients in the CCRT group received CCRT intervention, while those in the control group did not participate in any rehabilitation program. All patients in both the CCRT and control groups continued to receive psychotropic medication.

In addition, 29 healthy male individuals were selected using propensity score method in the study (33). Briefly, a logistic regression model was used to evaluate the covariates of the 40 enrolled patients, including age, marriage, and educational level, to find the closest propensity score, on which matching was performed among the healthy individuals within a prespecified range. Individuals who do not match within the restricted scope were excluded from this study.

### Computerized cognitive remediation therapy

The cognitive remediation therapy used in this study was a restorative-based cognitive training by rehearsal learning approach, which was delivered by the computer software in Chinese (CCRT-v1.0 system, No:2012SR085132, Kangze Medical Technology Co. Ltd, Guangzhou, China). The software was derived from the English version of the Frontal/Executive Function Program (34), and was developed to treat patients with psychosis with cognitive impairment in the People’s Republic of China (14). In this study, cognitive domains targeted by CCRT were classified according to the MATRICS Consensus domains: attention (4 tasks), working memory (8 tasks), speed of processing (cognitive flexibility, 6 tasks), reasoning and problem solving (10 tasks), and social cognition (2 tasks), with the exception of verbal learning and visual learning (35, 36). Each task in CCRT was rated based on their own difficulty. The therapist, who received initial training including CCRT theory, operation of CCRT software system, and evaluation for the difficulty of tasks, evaluated the difficulty of cognitive tasks and the actual performance scores of participants, including performance changes based on task complexity and individual response levels. To keep cognitive exercise challenging and engaging, the difficulty of the task will be dynamically adjusted when the accuracy rate reaches 80%, and each participant entailed practice at their own pace in personalized cognitive exercises. To produce meaningful effects of the intervention, the treatment program spanned for 8 weeks, and was conducted 5 times a week with 40-min session each time (i.e., 40 sessions). During each 40-min session, participants completed 8 tasks randomly selected from the cognitive modules: one task each from the attention and social cognition modules, and two tasks each from the cognitive flexibility, working memory, and problem-solving modules. The training time for each task was 5 minutes. However, the intervention program did not include self-generating strategies that promote cognition and problem-solving, as well as transferring cognitive skills to real life functioning.

### Assessments

#### Clinical symptoms

The Positive and Negative Syndrome Scale (PANSS) evaluates the severity of psychopathology in patients with schizophrenia through thirty-three items, covering positive, negative, and general symptoms (37). Anxiety and depression were assessed by the Hamilton Anxiety Rating Scale (HARS) (38) and Hamilton Depression Rating Scale (HDRS) (39), respectively. Cognitive impairment was estimated by Montreal Cognitive Assessment (MoCA) and Repeatable Battery for the Assessment of Neuropsychological Status (RBANS). MoCA consists of 13 tasks covering seven cognitive domains, including delayed recall, language, visuospatial/executive function, naming, attention, abstraction, and orientation. When the patient’s educational years did not exceed 12 years, the total MoCA score increased by 1 point (40). RBANS is composed of 12 subtests, divided into five cognitive domains: immediate memory, visuospatial, language, attention and delayed memory (41).

### Measurement of serum neurotrophic factors

10 ml peripheral blood was collected from the antecubital vein of each patient after clinical symptoms were assessed on the day of enrollment, 4 weeks, and 8 weeks. The blood sample was separated by centrifuge (Kehua, KHB-80) at 3000 rpm for 15 min, and the supernatant was stored at -80°C. GDNF and BDNF were detected by human GDNF enzyme-linked immunosorbent assay kit (JYMBio, China, Cat No.: JYM0166Hu) and human BDNF enzyme-linked immunosorbent assay kit (JYMBio, China, Cat No.: JYM0186Hu), respectively. Serum levels of GDNF and BDNF were analyzed and calculated according to the defined instructions.

### Sample size

Referenced from Khan et al.’s study on cognitive remediation training in patients with chronic schizophrenia (42), we estimated a necessary size of 36.6 (18.3 per group) according to the calculation formula (n = $\frac{{\left(\text{Z}\frac{\text{α}}{2}+\text{Zβ}\right)}^{2}\times (\delta_{1}^{\;2}+\delta_{2}^{\;2})}{{\left(\text{μ}1-\text{μ}2\right)}^{2}}$ , wherein $\text{Z}\frac{\text{α}}{2}$ and Zβ represent the critical value of bilateral standard under normal distribution corresponding to $\frac{\text{α}}{2}$ and 1-β, respectively, and δ and µ represent the standard deviation and mean value before or after intervention, respectively), with α = 0.05, β = 0.2, $\text{Z}\frac{\text{α}}{2}$ = 1.96, and Zβ = 0.84. During our enrollment process, 40 male long-term institutionalized inpatients with schizophrenia were included in this study.

### Statistical analysis

Data were analyzed using Statistical Package for Social Sciences (SPSS) version 26.0 for Windows and the SigmaPlot 13.0 software. The independent *Student’s t*- test was employed for comparing continuous variables between two groups, while the *chi*-square test was used for categorical variables. If the data failed by the Normality test, *Mann-Whitney* Rank Sum Test was used to compare the variables between two groups. Two Way Repeated Measures ANOVA was used to examine the interaction between two factors, and *Holm-Sidak* method was performed to analyze all pairwise multiple comparison procedures to identify significance between different groups. Finally, *Pearson* Product Moment correlation was used to test the correlation. All statistical analyses were two-tailed and a *p*-value of less than 0.05 was considered significant. Data were reported as mean ± SEM.

## Results

### Demographic and clinical characteristics

There were no significant differences between the schizophrenia (n = 40) and healthy control groups (n =29) in terms of age and education level (**Supplementary Table 1**). However, a higher percentage of patients with schizophrenia were unmarried compared to healthy controls (20% *vs*. 3.45%, χ2 = 4.061, *p* = 0.044). The participants in the CCRT and control groups were long-term institutionalized inpatients, and there was no difference in length of hospital stay between the CCRT and control groups. Additionally, the CCRT and control groups were matched at baseline in terms of age, marriage, education, duration of illness, family history, and dosage of psychotropic medication (**Supplementary Table 2**).

### Improvement in clinical symptoms

Compared to the control group, patients in the CCRT group exhibited significantly greater reductions in total PANSS score (F [2, 38] = 19.310, *p* < 0.001) and negative symptom score (F [2, 38] = 18.281, *p* < 0.001) (**Table 1**). A significant improvement in depression, as measured by HDRS, was also observed (F [2, 38] = 4.442, *p* = 0.015), but no significant changes were noted in anxiety symptoms (**Table 1**). These results were verified by the difference between control and CCRT groups in decline from baseline to end of 8-week treatment in PANSS total score, negative subscale, and HDRS (**Supplementary Table 3**).

**Table 1:** PANSS, HDRS and HARS at baseline and post-treatment in control and CCRT groups.

	Control group (n=20)			CCRT group (n=20)			*p*
	Baseline	Post 4 weeks	Post 8 weeks	Baseline	Post 4 weeks	Post 8 weeks	(T, G, T × G)
PANSS (Total)	80.90±2.11	80.90±2.11	80.90±2.11	77.30±2.68	75.90±2.72^***^	74.90±2.85^***^	< 0.001, 0.167, < 0.001
PANSS (Positive)	18.75±0.94	18.75±0.94	18.75±0.94	17.15±1.39	17.45±1.51	17.45±1.51	0.373, 0.425, 0.373
PANSS (Negative)	27.40±1.27	27.40±1.27	27.40±1.27	27.00±1.21	26.15±1.21^***^	25.65±1.24^***^	< 0.001, 0.523, < 0.001
PANSS (General)	34.75±2.09	34.75±2.09	34.75±2.09	33.15±1.72	32.30±1.79	31.80±1.81	0.207, 0.520, 0.711
HDRS (Total)	4.10±0.56	3.35±0.64	4.40±0.91	5.25±0.68	3.20±0.56^**^	2.75±0.43^***^	0.010, 0.769, 0.015
HARS (Total)	3.40±0.64	2.60±0.54	2.15±0.53	3.20±0.52	1.90±0.36^*^	1.85±0.27^*^	0.003, 0.459, 0.794

Values are presented as mean ± standard error. CCRT, computerized cognitive remediation therapy; Post, Post-treatment; PANSS, Positive and negative syndrome scale; HDRS, Hamilton depression rating scale; HARS, Hamilton anxiety rating scale; T, Time; G, Group; T×G, Interaction between time and group; ^*^ *p* < 0.05, ^**^ *p* < 0.01, ^***^ *p* < 0.001 *vs* Baseline in the same group.

### Improvement in cognitive function

Significant improvement in cognitive function was observed in the CCRT group, with higher total MoCA score (F [2, 38] = 14.121, p < 0.001) and RBANS score (F [2, 38] = 13.553, *p* < 0.001). Cognitive variables including delayed recall (list recall and figure recall), attention, and language showed marked improvement in the CCRT group compared to the control group (**Tables 2**, **3**). These results were confirmed by the value-added from baseline to the end of 8-week CCRT intervention (**Supplementary Table 4**). A significant association was found between the reduction in negative symptoms and the improvement in list recall (*p* = 0.044, **Supplementary Table 5**), suggesting that the improvement of memory ability contributes to alleviation of symptoms.

**Table 2:** MoCA and its subscales at baseline and post-treatment in control and CCRT groups.

	Control group (n=20)			CCRT group (n=20)			*p*
	Baseline	Post 4 weeks	Post 8 weeks	Baseline	Post 4 weeks	Post 8 weeks	(T, G, T×G)
MoCA (Total)	21.80±1.18	22.25±1.03	20.80±1.23	22.45±0.88	24.80±0.64^***^	25.95±0.52^***/###^	0.002, 0.033, <0.001
Delayed recall	1.95±0.34	2.45±0.41	1.45±0.29	1.50±0.38	2.45±0.41	2.65±0.34^*#^	0.060, 0.524, 0.022
Language	2.45±0.16	2.00±0.24	2.10±0.20	2.20±0.17	2.45±0.21	2.75±0.09^#^	0.466, 0.145, 0.017
Visuospatial/Executive	2.55±0.31	3.15±0.27	2.90±0.28	3.35±0.29^#^	3.60±0.22	3.90±0.21^#^	0.020, 0.026, 0.294
Naming	2.95±0.05	2.70±0.15	2.80±0.16	2.80±0.12	3.00±0.00	3.10±0.10	0.568, 0.167, 0.033
Attention	5.00±0.38	4.75±0.33	4.90±0.39	5.10±0.26	5.75±0.16^*/#^	5.85±0.10^**^/^#^	0.172, 0.071, 0.017
Abstraction	1.40±0.16	1.55±0.15	1.35±0.19	1.65±0.13	1.75±0.14	1.80±0.09	0.571, 0.074, 0.539
Orientation	5.40±0.23	5.40±0.23	5.20±0.26	5.65±0.18	5.75±0.14	6.00±0.00^##^	0.876, 0.041, 0.150

Values are presented as mean ± standard error. CCRT, computerized cognitive remediation therapy; MoCA, Montreal Cognitive Assessment; T, Time; G, Group; T×G, Interaction between time and group; ^*^ *p* < 0.05, ^**^ *p* < 0.01, ^***^ *p* < 0.001 *vs* Baseline in the same group; ^#^ *p* < 0.05, ^##^ *p* < 0.01, ^###^ *p* < 0.001 *vs* Control group in the same time.

**Table 3:** RBANS and its items at baseline and post-treatment in control and CCRT groups.

	Control group (n=20)			CCRT group (n=20)			*p*
	Baseline	Post 4 weeks	Post 8 weeks	Baseline	Post 4 weeks	Post 8 weeks	(T, G, T×G)
RBANS (Total)	63.80±3.11	66.55±3.16	66.70±3.09	64.45±2.07	72.75±2.97^***^	79.55±2.47^**^/^##^	<0.001, 0.091, <0.001
Immediate memory	51.85±3.01	62.35±4.54^**^	58.75±3.88^*^	54.55±2.85	61.20±4.35^*^	71.90±3.37^***^/^#^	<0.001, 0.302, 0.003
List learning	11.60±1.64	15.65±1.84^*^	14.10±1.46	15.00±1.43	16.25±1.81	20.25±1.52^***/^#^^	<0.001, 0.107, 0.012
Story memory	7.10±1.18	8.80±1.76	9.00±1.44	6.85±0.99	9.40±1.19^*^	12.20±0.97^***^	<0.001, 0.467, 0.056
Visuospatial	81.95±4.08	77.1±3.53	80.5±3.08	86.35±3.54	93.10±2.46^*/^##^^	95.00±2.95^**/^##^^	0.178, 0.007, 0.009
Figure copy	15.90±0.78	14.70±0.89	15.85±0.83	16.05±0.76	18.15±0.19^**/^###^^	18.15±0.28^**/^#^^	0.081, 0.021, 0.002
Line orientation	14.35±0.76	13.10±0.76	12.40±0.90	15.55±0.56	15.05±0.72	15.75±0.66	0.095, 0.052, 0.065
Language	82.35±2.33	81.65±3.42	78.50±3.55	81.00±3.05	86.45±1.88	88.50±2.02^**/^#^^	0.365, 0.195, 0.007
Picture naming	9.65±0.15	9.35±0.35	9.25±0.40	9.65±0.43	9.90±0.06	9.95±0.05	0.976, 0.199, 0.275
Semantic fluency	13.75±0.92	14.15±1.09	13.05±0.99	13.95±1.08	14.55±1.00	15.50±1.06	0.724, 0.415, 0.184
Attention	76.10±3.94	75.00±3.54	80.55±2.87	78.65±4.01	83.85±4.08	87.95±2.77^**^	0.004, 0.170, 0.278
Digit span	10.65±0.66	10.30±0.66	11.10±0.56	11.10±0.70	11.45±0.77	12.60±0.61^*^	0.032, 0.210, 0.449
Coding tasks	23.05±2.96	22.90±3.21	23.80±3.17	27.55±2.68	31.05±2.79	30.60±2.00	0.246, 0.092, 0.329
Delayed memory	60.60±4.17	67.60±3.63	66.60±3.98	57.20±3.20	68.65±4.11^***^	77.60±3.75^***/^#^^	<0.001, 0.558, 0.002
List recall	2.65±0.48	2.70±0.53	2.95±0.61	1.85±0.52	2.95±0.60^*^	4.40±0.61^***^	<0.001, 0.665, 0.009
List recognition	15.40±0.61	16.55±0.51	15.55±0.87	15.55±0.56	16.35±0.68	17.10±0.48	0.149, 0.449, 0.233
Story recall	3.75±3.46	4.15±0.75	4.75±0.85	3.85±0.76	4.85±0.68	6.40±0.57^***^	0.002, 0.365, 0.267
Figure recall	8.25±1.07	9.95±1.22	9.25±1.30	6.65±1.03	10.60±1.23^***^	13.00±0.98^***/^#^^	<0.001, 0.540, <0.001

Values are presented as mean ± standard error. CCRT, computer cognitive remediation therapy; RBANS, Repeatable battery for the assessment of neuropsychological status; T, Time; G, Group; T×G, Interaction between time and group; ^*^ *p* < 0.05, ^**^ *p* < 0.01, ^***^ *p* < 0.001 *vs* Baseline in the same group; ^#^ *p* < 0.05, ^##^ *p* < 0.01, ^###^ *p* < 0.001 *vs* Control group in the same time.

### Recovery in serum GDNF levels

**Figure 1:**
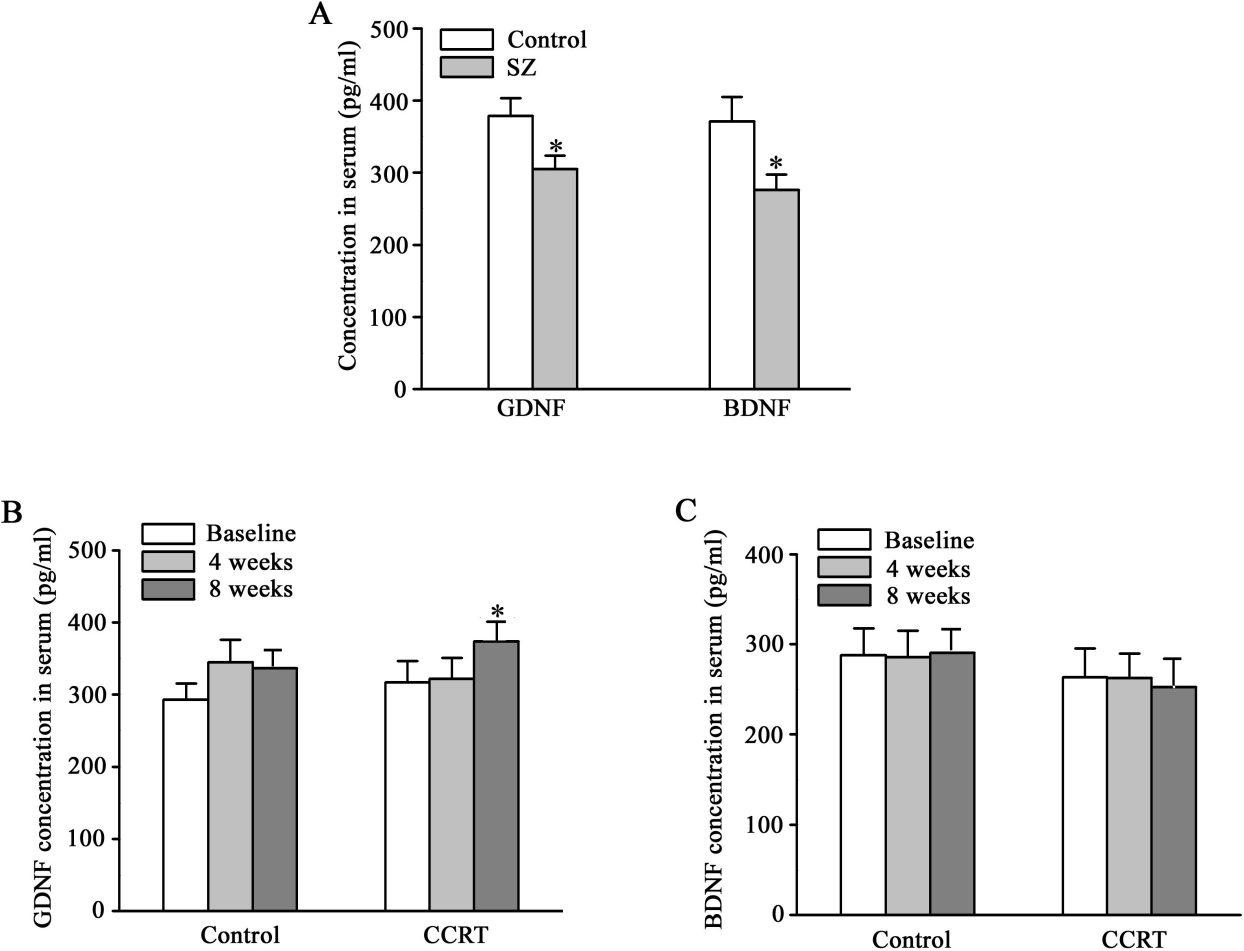
Effects of CCRT on serum levels of GDNF and BDNF in patients with schizophrenia. Serum levels of glial cell line-derived neurotrophic factor (GDNF) and brain-derived neurotrophic factor (BDNF) (pg/ml) in the subjects with schizophrenia (n = 40) and healthy controls (n = 29) **(A)**. Serum levels of GDNF (pg/ml) at baseline, 4 weeks and 8 weeks in CCRT (n = 20) and control groups (n = 20) **(B)**. Serum levels of BDNF (pg/ml) at baseline, 4 weeks and 8 weeks in CCRT (n = 20) and control groups (n = 20) **(C)**. ^*^ *p* < 0.05 *vs* control group.

Serum levels of GDNF and BDNF were significantly lower in patients with schizophrenia compared to healthy controls at baseline (GDNF: 304.838 ± 18.236 pg/ml vs. 378.534 ± 24.397 pg/ml, *p* = 0.012; BDNF: 275.821 ± 21.447 pg/ml vs. 370.983 ± 33.849 pg/ml, *p* = 0.010). At the end of 8-week CCRT intervention, GDNF levels significantly increased in the CCRT group compared to the control group (*p* = 0.033), while the BDNF levels did not show significant changes (**Figure 1**).

## Discussion

The randomized controlled trial demonstrated that CCRT alleviated psychiatric symptoms and cognitive deficits in male long-term institutionalized inpatients with schizophrenia. Specifically, CCRT was associated with a significant reduction in negative symptoms and improvement in memory recall. These findings suggest that cognitive impairment and negative symptoms share a common biological basis, and targeting cognitive deficits through cognitive remediation can have broader therapeutic benefits in patients with schizophrenia.

Schizophrenia is a chronic psychiatric disorder with multiple psychopathological symptoms, and cognitive remediation has emerged as a unique method for treating cognitive deficits, one of the core features in schizophrenia (43). A 2-year follow-up study confirmed that CR has a positive impact on cognitive functions in patients with schizophrenia, and indicated that CR, including self-generating strategies and transfer, may enable participants to acquire, practice and master essential occupational skills (44). Furthermore, participants who received CR intervention were likely to be employed at 5-year follow-up (45). A core feature of CR is cognitive exercise, where participants engage repetitively with stimuli to sustain the activation of relevant neuronal networks (46). It has been hypothesized that the number of cognitive exercise sessions completed would be directly related to positive treatment outcomes (47). Individual-level data from the National Institute of Mental Health Database of Cognitive Training and Remediation Studies verified that more CR sessions led to greater improvement in cognitive outcomes (48). In chronic schizophrenia, a CCRT intervention consisting of 60 individual 45-minute sessions, conducted 5 times per week for 12 weeks, improved cognitive function and social skills as measured by PANSS, Wisconsin Card Sorting Test, and Social Functioning Scale for Psychiatric Inpatients (49). One meta-analysis found that the average length of cognitive exercise was 32.2 hours, provided across 16.7 weeks (15). In chronic schizophrenia patients with cognitive impairment, 8 weeks of aerobic exercise did not improve cognitive performance and negative symptoms, while aerobic exercise combined with CCRT significantly improved cognitive ability and negative symptoms, indicating the impact of 8-week CCRT on cognitive function and negative symptoms (28). However, it is unclear whether the observed improvement in cognitive function and negative symptoms in the previous study was completely attributed to CCRT treatment. In the current study, 40 long-term inpatients with schizophrenia were included, of which 20 patients only received CCRT intervention (**Supplementary Figure 1**). The intervention includes 40 individual 40-min sessions administered 5 times a week, over an 8-week period, to demonstrate the efficiency of CCRT in clinical symptoms, cognitive functioning, and serum levels of GDNF and BDNF.

The mixed-design ANOVA showed a time effect and a time × group interaction in total and negative PANSS scores (**Table 1**). These results indicated that group type, in this case, patients who underwent CCRT and those who did not receive the treatment, significantly affected the severity of psychopathology in schizophrenia. Compared to the control group, participants in the CCRT group reported significant improvement in psychiatric symptoms, especially in the negative component (**Table 1**). To confirm this finding, we compared the decrement from baseline to the end of 8-week treatment in PANSS scores between the control and CCRT groups. A significant difference was observed in total and negative PANSS scores between the control and CCRT groups (**Supplementary Table 3**). Taken together, the methodology of CCRT in our study verified the concept that negative symptoms have been proposed as a new target for cognitive remediation (13, 50–52).

More than 60% of patients with schizophrenia suffer from depression, especially chronic schizophrenia (53, 54), and 52.7% of patients suffer from anxiety symptoms that have reached the clinical level (55). Among Chinese male inpatients with schizophrenia, the prevalence of depression was 62.2% using depression scales unique to non-psychotic patients, and 41.8% with the Chinese Calgary Depression Scale for Schizophrenia (56, 57). Our results suggest that CCRT alleviated depressive symptoms by the mixed-design ANOVA and independent *Student’s t*-test (**Table 1**, **Supplementary Table 3**). A previous study reported similar results for depression, revealing that CCRT improved mood by targeting at executive dysfunction (58). Indeed, neuroimaging findings indicated that the pathogenesis of depressive symptoms in schizophrenia could be linked to the altered functioning in the dorsolateral prefrontal cortex (DLPFC) (59, 60). In chronic schizophrenia, poor executive performance, as measured by the Wisconsin Card Sorting Test (WCST), has been linked to lower volume of the left DLPFC (61, 62). This finding aligns with functional MRI studies, which showed reduced activity of the left DLPFC during WCST performance (63). Furthermore, anodal stimulation of the left DLPFC has been proven to be an effective protocol for treating depressive symptoms in both schizophrenia and depression (64–66). Therefore, targeting executive functions, such as problem-solving and cognitive flexibility, in the current study may contribute to alleviating depressive symptoms in patients with schizophrenia.

Cognitive impairment is considered as a core component of schizophrenia, with 98% of patients showing cognitive decrement compared to their premorbid state (67). MoCA, as a bedside cognitive screening tool for patients with schizophrenia in the fast-paced clinical setting, has sufficient concurrent effectiveness. Analysis revealed that MoCA scores of 25 or above are normal, while patients with scores below 23 are likely to have severe cognitive impairment (68). In the present study, the average MoCA score of patients with schizophrenia was 22.45, classified as severe cognitive impairment. At the end of 8-week CCRT, the average score of the patients was 25.95 (**Table 2**), indicating a potential recovery to ‘normal’ cognitive function (68). The cognitive domains improved by CCRT are delayed recall, language, and attention (**Table 2**, **Supplementary Table 4**). However, 6-week CCRT did not produce benefits for cognitive functions, as assessed by the composite score of the Measurement and Treatment Research to Improve Cognition in Schizophrenia (MATRICS) Consensus Cognitive Battery, despite the intervention’s efficiency in reducing negative symptoms (51). To address the positive impact of the CCRT on cognitive deficits in schizophrenia, RBANS, which has demonstrated good reliability, sensitivity, and specificity for the cognitive deficits associated with schizophrenia (69, 70), was utilized in the study. The methodology of CCRT in the study significantly increased the average score of RBANS in patients with schizophrenia from 64.45 to 79.55 (**Table 3**). Furthermore, CCRT improved the performance in five subtests-story memory, figure copy, line orientation, list recall, and figure recall, as well as four indexes: immediate memory, visuospatial ability, language, and delayed memory (**Table 3**, **Supplementary Table 4**).

While previous data demonstrated a correlation between MoCA score and PANSS negative symptoms subscale (71), we did not replicate this finding. Instead, our data showed a relationship between the decrement in PANSS negative symptoms subscale and the increment in the score of list recall test in RBANS (**Supplementary Table 5**). Comparing cognitive functions of responders with those of non-responders to treatment, significant differences also appeared in list recall components of Korean version of Memory Assessment Scales (72). In Chines Han patients with schizophrenia, Pearson’s analysis, especially after performing Bonferroni corrections, verified that PANSS negative sub-score was negatively correlated with the RBANS total and memory index scores (73). The dopamine hypothesis is the most influential theory in the neurochemical basis of schizophrenia, which suggests that fundamental dysregulation of the dopamine system is the cause of symptoms in schizophrenia (74). Reward learning, which is used to identify the cognitive processes responsible for adapting behavior, has been linked to the dopamine system in the brain. Reward learning abnormalities, induced by dysregulation of the dopamine system, are thought to influence negative symptoms and cognitive deficits in schizophrenia. Therefore, reward learning is the pathway for the effect of CR on negative symptoms and cognitive deficits (75). In addition, the beneficial effect of CR on negative symptoms in individuals with schizophrenia has confirmed the causality of the cognition-negative symptom relation (18, 76). However, little is known about the reasons for the relationship between reduction in negative symptoms and improvement in memory recall by cognitive remediation. The features of alexithymia, such as poverty of thought and expression, blunting of affect, and alogia, are similar to the characteristics of negative symptoms, but not positive symptoms in schizophrenia (77). In addition, the link between alexithymia and a broad range of impaired neurocognition has been ascertained (78). A previous study considered that alexithymia may be a link between cognitive and negative symptoms in schizophrenia (79). A recent discovery confirmed the theoretical viewpoint that alexithymia played a mediating role in the pathway from cognitive impairment to negative symptoms (78). Therefore, alexithymia might play a role in CCRT-induced improvement of negative symptoms by targeting memory performance, particularly list recall.

BDNF and GDNF, the most extensively investigated neurotrophins related to psychotic disorders, play a key role in cognitive processes (80, 81). A previous study showed that serum levels of BDNF and GDNF were markedly lower in the first-episode drug-naïve patients with schizophrenia than in healthy controls (82). Our data replicated this finding (**Figure 1A**), although a report showed differences in BDNF levels between patients with schizophrenia and healthy controls, rather than GDNF levels (83). After neuroplasticity-based cognitive training, serum levels of BDNF are significantly increased in patients with chronic schizophrenia (27). However, Rafael Penades et al. found that serum BDNF levels were augmented during the cognitive remediation only for the Val/Val group not for the Met carriers in the Hospital Clinic of Barcelona (26). Among Asians, the proportion of Met allele carriers of BDNF gene (41%) is significantly higher than that of Caucasians (18%) (84), and Met allele is a risk factor for schizophrenia (85). Therefore, the current trial did not replicate the previous finding of elevated serum BDNF levels after cognitive remediation in Chinese patients. The analysis of GDNF levels of African Americans and Caucasians did not show ethnicity-related difference (86). Furthermore, serum GDNF levels in patients with deficit schizophrenia who performed better in cognitive tests were higher than the average (23, 83, 87). In the present study, increase in serum GDNF levels after CCRT (**Figure 1**) suggests that GDNF may serve as a potential biomarker for the response to cognitive remediation in schizophrenia. While BDNF has been widely studied as a marker for cognitive recovery, our findings did not show significant changes in BDNF levels, which may be due to genetic variations affecting BDNF expression in different populations (85). Further research is needed to investigate the role of GDNF and its interaction with other neurotrophic factors in cognitive and clinical improvement in patients with schizophrenia.

## Limitations

Of note, this study has several limitations. Firstly, this study used a small sample of participants (both control and CCRT groups), all of whom were long-term institutionalized patients with stable schizophrenia, given the research setting. However, the sample size met the minimum theoretical requirements for the study. In this program, the stability of symptoms and medication dosage in patients with schizophrenia is necessary to verify the effects of CCRT on clinical symptoms and serum levels of neurotrophic factors. The impact of physiological cycle on symptom stability and antipsychotic drug dosage has been confirmed (88, 89), so female patients were excluded from this trial. Therefore, the sample was comprised of long-term institutionalized male inpatients with stable symptoms under standardized medication condition, and the final proportion of inpatients included was approximately 32.2% (40/124). As such, it is unclear to what extent the results of this study are applicable to female patients with schizophrenia. Further, CCRT intervention, in this study, focused on cognitive exercise, but did not include facilitation of cognitive, problem-solving self-generating strategies, or transfer of learning into real life functioning. Therefore, no functional outcome measurement was administered. In addition, executive functioning (e.g., problem solving, cognitive flexibility), which are cognitive domains commonly affected in individuals with schizophrenia and measured by MATRICS Consensus Battery, could not be assessed using either MoCA or RBANS in this study.

## Conclusions

In conclusion, 8-week cognitive remediation training promoted the alleviation of negative symptoms, depressive symptoms, and cognitive deficits in male inpatients with stable schizophrenia. The improvement of negative symptoms was associated with the enhancement of cognitive function, particularly list recall. Additionally, GDNF might be involved in the specific effects of CCRT on patients with schizophrenia. Future studies should investigate the long-term effects of CCRT and its applicability in female patients and other populations.

## Data Availability

The raw data supporting the conclusions of this article will be made available by the authors, without undue reservation.
