# Supplementary material for: Enhanced computerized cognitive remediation therapy improved cognitive function, negative symptoms, and GDNF in male long-term inpatients with schizophrenia
Source: Front Psychiatry. 2025 Jan 16;15:1477285. doi: 10.3389/fpsyt.2024.1477285 (PMC11780405; doi:10.3389/fpsyt.2024.1477285)
Supplement: Supplementary file 1 [file DataSheet1.zip › Supplementary Figure 1.DOCX]

Enrollment

Male inpatients with schizophrenia (n=124)

Excluded (n=84)

Declined to participate (n=6)

Enrolled in other studies (n=14)

Not meeting the criteria of age (n=14)

Acute phase of schizophrenia (n=45)

Instability of illness (n=5)

Male healthy control (n=46)
()

Excluded (PSM, n=17)

- Age (n=10)
- Education (n=7)

Male healthy control (n=29)

Randomized (n=40)

Comparison in GDNF and BDNF

CCRT group

Control group

Allocation

Allocated to intervention (n=20):

Routine medication

CCRT intervention

Allocated to intervention (n=20):

Routine medication

Did not receive CCRT

Follow-up

**Supplementary Figure 1.** Enrollment, allocation of groups and follow-up processes are presented with CONSORT flow-diagram.

CONSORT, consolidated standards of reporting trials; CCRT, computerized cognitive remediation therapy; GDNF, glial cell line-derived neurotrophic factor; BDNF, brain-derived neurotrophic factor.

Analyzed (n=20)

Excluded from analysis (n=0)

Analyzed (n=20)

Excluded from analysis (n=0)

Analysis

Lost to follow-up (n=0)

Continued intervention (n=20)

Lost to follow-up (n=0)

Continued intervention (n=20)
