# Supplementary material for: Enhanced computerized cognitive remediation therapy improved cognitive function, negative symptoms, and GDNF in male long-term inpatients with schizophrenia
Source: Front Psychiatry. 2025 Jan 16;15:1477285. doi: 10.3389/fpsyt.2024.1477285 (PMC11780405; doi:10.3389/fpsyt.2024.1477285)
Supplement: Supplementary file 1 [file DataSheet1.zip › Supplementary Table 1.DOCX]

**Supplementary Table 1**

Demographic characteristics of healthy control and schizophrenia

Variables Healthy control Schizophrenia *p*

(n=29) (n=40)

Age (year) 48.280±2.114 47.530±2.048 0.692

Marriage 1/28/0 8/32/0 0.044^*a^

(Unmarried/Married/Divorced)

Educational level (year) 10.140±0.328 10.750±0.429 0.293

Values are presented as mean ± standard error. ^*^, *p* < 0.05 *vs* Healthy control; ^a^, Chi-square analysis.
