# Supplementary material for: Enhanced computerized cognitive remediation therapy improved cognitive function, negative symptoms, and GDNF in male long-term inpatients with schizophrenia
Source: Front Psychiatry. 2025 Jan 16;15:1477285. doi: 10.3389/fpsyt.2024.1477285 (PMC11780405; doi:10.3389/fpsyt.2024.1477285)
Supplement: Supplementary file 1 [file DataSheet1.zip › Supplementary Table 2.DOCX]

**Supplementary Table 2**

Demographics and medication of control and CCRT groups in schizophrenia

| Variables | Control group  (n=20) | CCRT group  (n=20) | *p* |
| --- | --- | --- | --- |
| Age (year) | 48.200±2.114 | 46.850±2.048 | 0.649 |
| Marriage  (Unmarried/Married/Divorced) | 6/14/0 | 2/18/0 | 0.114^a^ |
| Educational level (year) | 10.850±0.586 | 10.650±0.641 | 0.819 |
| Duration of illness (year) | 21.938±2.308 | 21.533±2.069 | 0.897 |
| Length of hospital stay (year) | 9.700±4.014 | 7.600±3.830 | 0.099 |
| Family history (yes/no) | 6/14 | 4/16 | 0.465^a^ |
| Relative antipsychotic dosage | 2.021±0.243 | 1.898±0.214 | 0.708 |
| Relative sleeping dosage | 0.186±0.089 | 0.040±0.027 | 0.126 |

Values are presented as mean ± standard error. CCRT, computerized cognitive remediation therapy; ^a^, Chi-square analysis.
