# Supplementary material for: Enhanced computerized cognitive remediation therapy improved cognitive function, negative symptoms, and GDNF in male long-term inpatients with schizophrenia
Source: Front Psychiatry. 2025 Jan 16;15:1477285. doi: 10.3389/fpsyt.2024.1477285 (PMC11780405; doi:10.3389/fpsyt.2024.1477285)
Supplement: Supplementary file 1 [file DataSheet1.zip › Supplementary Table 3.DOCX]

**Supplementary Table 3**

Decrease in scores of psychiatric symptoms at end of 8 week-treatment in groups

| Variables | Control group  (n=20) | | CCRT group  (n=20) | | *t* | | *p* | |
| --- | --- | --- | --- | --- | --- | --- | --- | --- |
| PANSS (Total) | 0.000±0.000 | 2.400±0.515 | | 47.965 | | < 0.001^***^ | |  |
| PANSS (Positive) | 0.000±0.000 | -0.300±0.300 | | 4.457 | | 0.324 | |  |
| PANSS (Negative) | 0.000±0.000 | 1.350±0.274 | | 64.454 | | < 0.001^***^ | |  |
| PANSS (General) | 0.000±0.000 | 1.350±0.436 | | 37.985 | | 0.004^**^ | |  |
| HDRS (Total) | -0.300±0.900 | 2.500±0.678 | | 0.646 | | 0.018^*^ | |  |
| HARS (Total) | 1.250±0.717 | 1.350±0.466 | | 0.636 | | 0.908 | |  |

Values are presented as mean ± standard error. CCRT, computerized cognitive remediation therapy. PANSS, Positive and negative syndrome scale; HDRS, Hamilton depression rating scale; HARS, Hamilton anxiety rating scale. ^*^, *p* < 0.05, ^**^, *p* < 0.01, ^***^*p* < 0.001 *vs* control group.
