# Supplementary material for: Enhanced computerized cognitive remediation therapy improved cognitive function, negative symptoms, and GDNF in male long-term inpatients with schizophrenia
Source: Front Psychiatry. 2025 Jan 16;15:1477285. doi: 10.3389/fpsyt.2024.1477285 (PMC11780405; doi:10.3389/fpsyt.2024.1477285)
Supplement: Supplementary file 1 [file DataSheet1.zip › Supplementary Table 4.docx]

**Supplementary Table 4**

Increase in scores of MoCA and RBANS at end of 8-week treatment in groups

| Variables | Control group  (n=20) | CCRT group  (n=20) | *t* | *p* |
| --- | --- | --- | --- | --- |
| MoCA(Total) | -1.000±0.668 | 3.500±0.626 | 4.911 | < 0.001^***^ |
| Delayed recall | -0.500±0.401 | 1.150±0.365 | 3.045 | 0.004^**^ |
| Language | -0.350±0.182 | 0.550±0.198 | 3.346 | 0.002^**^ |
| Visuospatial/executive | 0.350±0.293 | 0.550±0.235 | 0.533 | 0.597 |
| Naming | -0.150±0.167 | 0.300±0.179 | 1.839 | 0.074 |
| Attention | -0.100±0.261 | 0.750±0.270 | 2.264 | 0.029^*^ |
| Abstraction | -0.050±0.211 | 0.150±0.109 | 0.841 | 0.406 |
| Orientation | -0.200±0.236 | 0.35±0.182 | 1.846 | 0.073 |
| RBANS(Total) | 2.900±5.674 | 15.100±8.656 | 5.271 | < 0.001^***^ |
| Immediate memory | 6.900±1.789 | 17.350±3.142 | 2.891 | 0.006^*^ |
| List learning | 2.500±0.786 | 5.250±1.509 | 1.616 | 0.114 |
| Story memory | 1.900±0.760 | 5.350±1.172 | 2.468 | 0.018^*^ |
| Visuospatial | -1.450±3.138 | 8.650±2.717 | 2.433 | 0.020^*^ |
| Figure copy | -0.050±0.693 | 2.100±0.640 | 2.270 | 0.028^*^ |
| Line orientation | -1.950±0.494 | 0.200±0.367 | 3.491 | 0.001^**^ |
| Language | -3.850±2.668 | 7.500±2.782 | 2.945 | 0.005^**^ |
| Picture naming | -0.400±0.293 | 0.300±0.429 | 1.345 | 0.187 |
| Semantic fluency | -0.700±1.275 | 1.550±0.759 | 1.516 | 0.138 |
| Attention | 4.450±3.267 | 9.300±3.192 | 1.062 | 0.295 |
| Digit span | 0.450±0.670 | 1.500±0.678 | 1.100 | 0.278 |
| Coding tasks | 0.750±1.704 | 3.050±1.464 | 1.024 | 0.313 |
| Delayed memory | 6.000±2.214 | 20.400±2.851 | 3.989 | < 0.001^***^ |
| List recall | 0.300±0.487 | 2.550±0.568 | 3.004 | 0.005^**^ |
| List recognition | 0.150±0.941 | 1.550±0.630 | 1.236 | 0.224 |
| Story recall | 1.000±0.664 | 2.550±0.872 | 1.413 | 0.166 |
| Figure recall | 1.000±0.733 | 6.350±0.666 | 5.403 | < 0.001^***^ |

Values are presented as mean ± standard error. CCRT, computerized cognitive remediation therapy. MoCA, Montreal Cognitive Assessment；RBANS, Repeatable Battery for the Assessment of Neuropsychological Status. ^*^, *p* < 0.05, ^**^, *p* < 0.01, ^***^*p* < 0.001 *vs* control group.
