# Supplementary material for: Enhanced computerized cognitive remediation therapy improved cognitive function, negative symptoms, and GDNF in male long-term inpatients with schizophrenia
Source: Front Psychiatry. 2025 Jan 16;15:1477285. doi: 10.3389/fpsyt.2024.1477285 (PMC11780405; doi:10.3389/fpsyt.2024.1477285)
Supplement: Supplementary file 1 [file DataSheet1.zip › Supplementary Table 5.DOCX]

**Supplementary Table 5**

Correlation between amelioration of psychiatric symptoms and upgradation of MoCA and RBANS at end of 8-week CCRT

|  | |  | | | PANSS | | |  | | |  | | HDRS  (Total)  Coef. *p* | | HARS  (Total)  Coef. *p* | | |  |
| --- | --- | --- | --- | --- | --- | --- | --- | --- | --- | --- | --- | --- | --- | --- | --- | --- | --- | --- |
|  |  | Total  Coef.  *p* | | | Positive  Coef. *p* | | | Negative  Coef. *p* | | | General  Coef. *p* | |  |  |  |  |  |  |
| MoCA (Total) | -0.204 | | 0.388 | -0.126 | | 0.596 | -0.161 | | 0.498 | -0.529 | | 0.825 | 0.235 | 0.318 | | 0.049 | 0.836 | |
| Delayed recall | 0.157 | | 0.509 | 0.123 | | 0.606 | 0.133 | | 0.576 | 0.017 | | 0.942 | 0.229 | 0.332 | | -0.200 | 0.397 | |
| Language | -0.092 | | 0.697 | -0.411 | | 0.071 | 0.138 | | 0.562 | 0.086 | | 0.717 | 0.029 | 0.902 | | 0.110 | 0.646 | |
| RBANS(Total) | -0.134 | | 0.573 | 0.255 | | 0.278 | -0.151 | | 0.524 | -0.238 | | 0.311 | 0.115 | 0.629 | | -0.342 | 0.139 | |
| Immediate memory | 0.122 | | 0.639 | 0.162 | | 0.496 | -0.161 | | 0.498 | -0.037 | | 0.875 | 0.109 | 0.647 | | -0.333 | 0.151 | |
| Visuospatial ability | -0.093 | | 0.694 | 0.142 | | 0.549 | -0.036 | | 0.877 | -0.185 | | 0.435 | -0.269 | 0.251 | | -0.190 | 0.422 | |
| Figure copy | -0.425 | | 0.062 | -0.008 | | 0.973 | -0.109 | | 0.646 | -0.426 | | 0.006^**^ | -0.170 | 0.475 | | -0.179 | 0.450 | |
| Language | -0.125 | | 0.600 | -0.128 | | 0.357 | 0.123 | | 0.607 | -0.074 | | 0.754 | 0.131 | 0.582 | | -0.196 | 0.408 | |
| Delayed Memory | 0.032 | | 0.891 | 0.214 | | 0.365 | -0.078 | | 0.743 | -0.059 | | 0.803 | -0.058 | 0.815 | | -0.408 | 0.073 | |
| List recall | 0.417 | | 0.067 | -0.050 | | 0.831 | 0.453 | | 0.044^*^ | 0.242 | | 0.304 | -0.092 | 0.700 | | -0.418 | 0.066 | |
| Figure recall | -0.285 | | 0.223 | 0.051 | | 0.830 | 0.064 | | 0.788 | -0.412 | | 0.071 | -0.207 | 0.382 | | -0.403 | 0.078 | |

RBANS, Repeatable Battery for the Assessment of Neuropsychological Status; CCRT, computerized cognitive remediation therapy; PANSS, Positive and negative syndrome scale; HDRS, Hamilton depression rating scale; HARS, Hamilton anxiety rating scale; Coef.: Correlation Coefficient; ^*^, *p* < 0.05; ^**^, *p* < 0.01.
